# Rapid metastasis of mediastinal solitary fibrous tumor: Report a case

**DOI:** 10.1097/MD.0000000000009307

**Published:** 2017-12-22

**Authors:** Yingming Xiang, Shaosong Tu, Fangbiao Zhang

**Affiliations:** Department of Cardiothoracic Surgery, Zhejiang University, Lishui Center Hospital, Lishui, Zhejiang, P.R. China.

**Keywords:** mediastinum, metastasis, solitary fibrous tumor, treatment

## Abstract

**Rationale:**

Mediastinal solitary fibrous tumors (SFTs) are rare mesenchymal neoplasms. Complete resection is considered as the effective treatment and the prognosis is quite good. Rapid metastasis after surgery is extremely rare.

**Patient concerns:**

In this case report we describe a 42-year-old man who present with a mediastinal malignant SFTs. Enhanced computed tomography of chest revealed a 4.5 × 4.0-cm mass in the anterior mediastinum.

**Diagnoses:**

The tumor is composed of massive proliferation of atypical spindle cells. Immunohistochemical staining for cluster of differentiation (CD) 34, CD99, and vimentin were strongly positive.

**Interventions:**

Due to the possibility that the tumor was malignant, a standard median sternotomy was performed under general anesthesia. The mediastinal tumor and the affected part of the pericardium and right upper lobe of the lung were completely resected.

**Outcomes:**

The patient underwent surgery and recovered uneventfully. After 2 months follow-up postoperation, there was recurrence in the chest wall and right middle lung. The patient refused any treatment and was dead after 2 months.

**Lessons:**

The present cases indicate that mediastinal SFTs should always be kept in mind for rapid metastasis. Once found, surgical intervention should be performed promptly. Due to the rapid metastasis, radiotherapy, and chemotherapy may be needed after surgery and long-term follow-up is required to monitor the metastasis of this type of tumor.

## 1 Introduction

Solitary fibrous tumors (SFTs) are rare nonepithelial neoplasms that most often involve the visceral pleura, which were initially described by Klemperer and Rabin in 1931.^[1]^ Since their discovery, SFTs have been identified in numerous extrapleural locations, including the lung, liver, thyroid, kidney, orbit, esophagus, pelvic, pancreas, omentum, head and neck, central nervous system, bladder, soft tissues of the extremities, and palatine tonsil.^[2]^ To our knowledge, mediastinal SFTs with lung and pericardium invasion are extremely rare and mostly grows slowly and usually found incidentally. After that, due to the tumor growth, such symptoms occur: cough, chest pain, and chest tightness.^[3]^ Complete resection is considered as the most effective and successful treatment of mediastinal SFTs and the prognosis is good. The present study presents a rare case of rapid metastasis in mediastinal malignant SFT with lung and pericardium invasion in a 42-year-old man, and reviews the previously reported cases in the literature.

## 2 Case presentation

**Figure 1:**
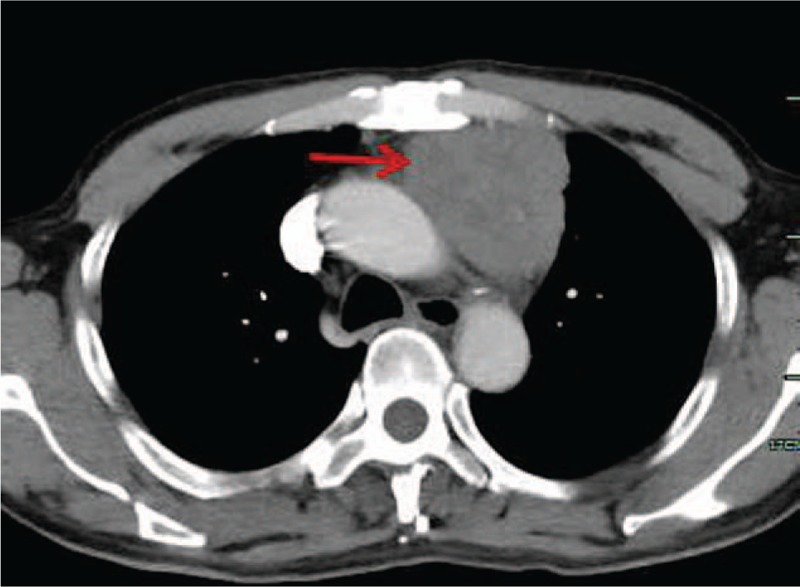
The contrast-enhanced CT revealed a 4.5 × 4.0 cm solid mass involving the anterior mediastinum (arrows).

**Figure 2:**
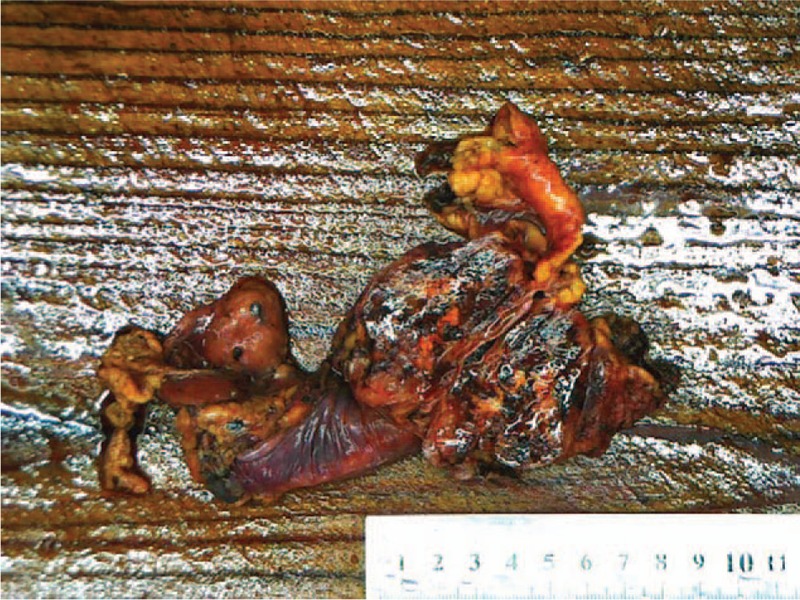
The tumor was solid and measured 6.0 × 4.5 × 3.5 cm.

**Figure 3:**
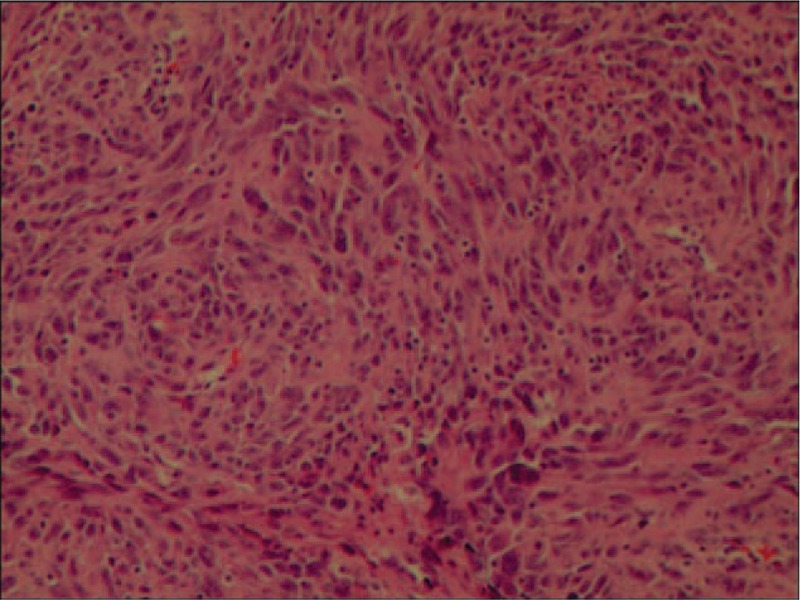
Histopathological examinations revealed that the mass consisting of massive proliferation of atypical spindle cells (100×).

**Figure 4:**
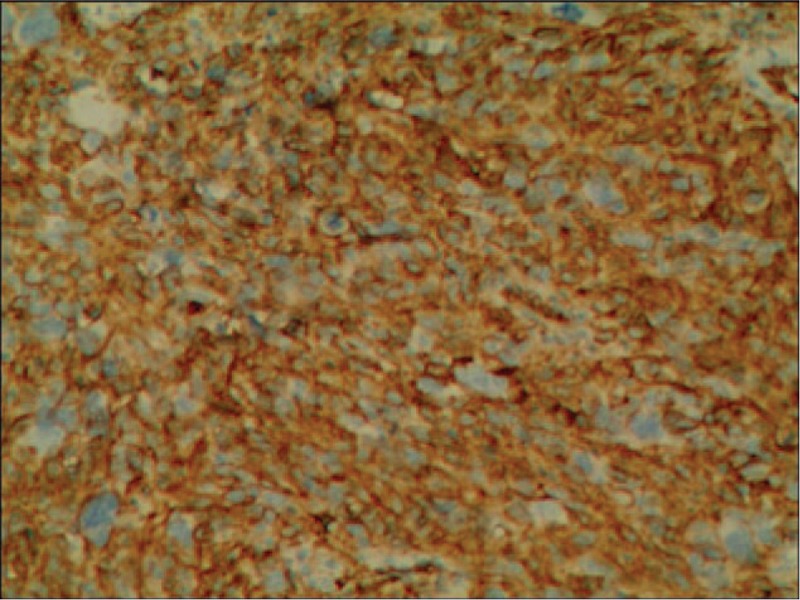
The immunohistochemical reactions for CD34 were positive (100×).

**Figure 5:**
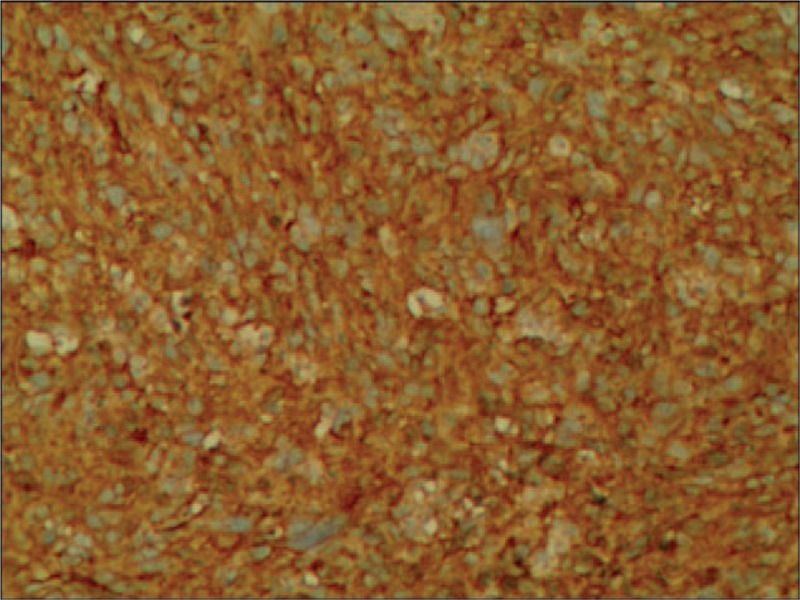
The immunohistochemical reactions for CD99 protein were positive (100×).

**Figure 6:**
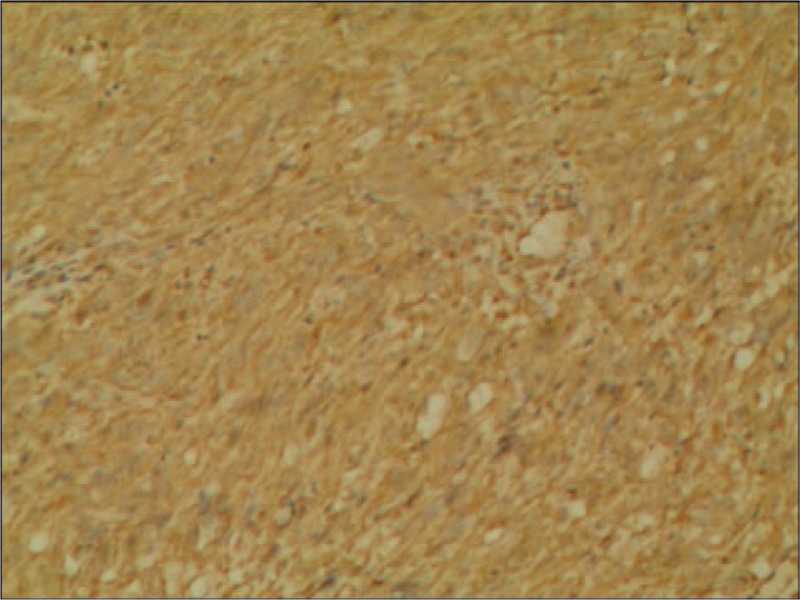
The immunohistochemical reactions for vimentin were positive (100×).

**Figure 7:**
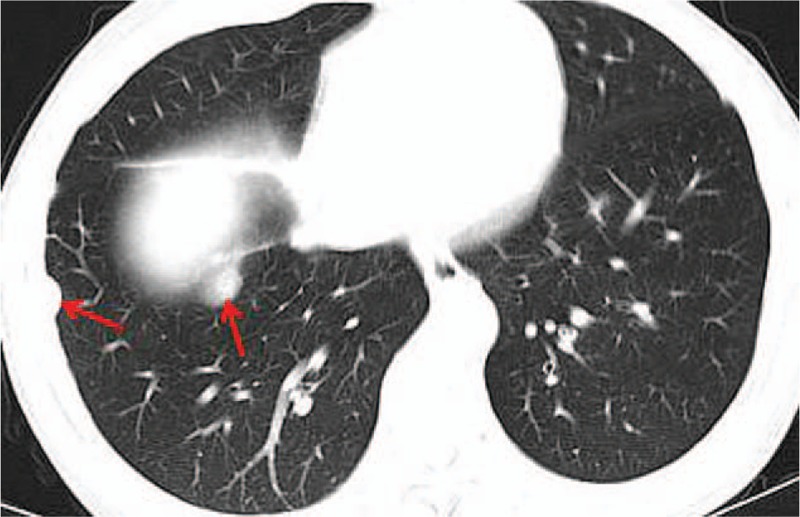
The contrast-enhanced CT revealed recurrence in the patient's chest wall and right middle lung (arrows).

A 42-year-old man referred to Lishui Center Hospital due to dry cough that had progressed over 12 months. The patient had a history of cigarette smoking and no history of hypertension, type II diabetes mellitus, coronary heart disease, hepatitis, obesity, or tuberculosis. An enhanced computed tomography (CT) scan (Brilliance iCT; Philips Healthcare, Amsterdam, The Netherlands) of the chest revealed a 4.5 × 4.0 cm solid mass involving the anterior mediastinum (Fig. 1). Further examinations, including routine blood [hemoglobin, 126 g/L (normal range, 120–160 g/L); red blood cell count, 3.95 × 10^12^/L (normal range, 3.5–5.5 × 10^12^/L); white blood cell count, 7200 cells/mL (normal range, 4000–10,000 cells/mL); platelet count, 1330 cells/mL (normal range, 1000–3000 cells/mL)], serum electrolyte [Na^+^, 141.0 mmol/L (normal range, 137–147 mmol/L); K^+^, 4.0 mmol/L (normal range, 3.5–5.3 mmol/L); Mg^2+^, 0.87 mmol/L (normal range, 0.64–1.25 mmol/L); Cl^−^, 105 mmol/L (normal range, 99–110 mmol/L); Ca^2+^, 2.10 mmol/L (normal range, 2.03–2.67 mmol/L); P^5+^, 0.96 mmol/L (normal range, 0.84–1.51 mmol/L)], glucose level [4.38 mmol/L (normal range, 3.9–6.1 mmol/L)] coagulation function [prothrombin time, 10.8 seconds (normal range, 10.5–14.0 seconds); activated partial thromboplastin time, 27.6 seconds (normal range, 23.5–36.0 seconds); thrombin time, 19.0 seconds (14.0–21 seconds); international normalized ratio, 0.91 (normal range, 0.8–1.2)], liver function [glutamic–pyruvic transaminase, 20 U/L (normal range, 9–50 U/L); glutamic oxalacetic transaminase, 21 U/L (normal range, 15–40 U/L)], renal function [creatinine, 67 μmol/L (normal range, 55–105 μmol/L); urea nitrogen, 7.3 mmol/L (normal range, 2.8–8.2 mmol/L)], electrocardiogram [heart rate, 83 beats per minute (normal range, 60–100 beats per minute)], were all within normal limits. Distant metastasis or dissemination was not found during ultrasound (US) of the abdomen, radionuclide bone scanning, and magnetic resonance imaging (MRI) of the head. Due to the possibility that the tumor was malignant, a standard median sternotomy was performed under general anesthesia. The mediastinal tumor and the affected part of the pericardium and right upper lobe of the lung were completely resected. On macroscopic examination, the tumor was firm and measured 6.0 × 4.5 × 3.5 cm (Fig. 2). The cut surface was gray. Resected tissue specimens were formalin-fixed, paraffin-embedded and cut into 4-μm sections. Histopathological examinations using hematoxylin and eosin staining (Sinopharm Chemical Reagent Co., Ltd, Shanghai, China) revealed that the mass corresponded to an unencapsulated mesenchymatous neoplasm, consisting of massive proliferation of atypical spindle cells (Fig. 3). The diagnosis of a mediastinal malignant SFT was established by immunohistochemistry, which revealed a positive immunoreaction to CD34 (Fig. 4), CD99 (Fig. 5), and vimentin (Fig. 6). Smooth muscle actin (SMA), CK, S-100, CD117, and myoglobin were negative. The patient was discharged on the thirteenth day postsurgery, following an uneventful recovery. Recurrence to the patient's chest wall and right middle lung were also identified after 2 months (Fig. 7). But the patient refused any treatment such as chemotherapy or radiation therapy and was dead after approximately 4 months after the initial diagnosis.

## 3 Discussion

SFTs are rare mesenchymal neoplasms that occur in the pleura, and are occasionally located at the mediastinum. The origin of SFTs was initially derived from the mesothelial cells. However, in recent years, based on immunohistochemistry and electron microscopy, it has become well established that SFTs arise from CD34-positive dendritic mesenchymal cells.^[3]^ In addition to the pleura, SFTs can also occur in the any body parts, such as lung, liver, thyroid, kidney, orbit, esophagus, pelvic, pancreas, as well as mediastinum.^[2]^ To date, most existing literature of mediastinal SFTs are limited to case reports.

SFTs are usually found incidentally and observed in middle-aged adults between 20 and 70 years with no sex predilection.^[4]^ Dependent on tumor location and size, patients present with different symptoms, including cough, chest pain, and chest tightness.^[3]^ In our case, the patient presented with dry cough that had progressed over 12 months. Of note, some patients might have tumor-associated symptoms, such as hypoglycemia, which is called Doege–Potter syndrome (DPS).^[5]^

Because of the lack of specific imaging features, mediastinal SFTs are relatively difficult to distinguish from other tumors before surgery. According to the study of England et al.^[6]^ The criteria for the diagnosis of malignant SFTs should include: high cellularity with crowded or overlapping nuclei; nuclear pleomorphism; high mitotic activity, with >4 mitotic figures per 10 high-power fields; and pleomorphic giant cells and abnormal mitotic activity.^[6]^ The differential diagnosis of mediastinal SFTs includes numerous malignant and benign tumors, including malignant mesothelioma, peripheral nerve sheath tumors, spindle cell thymoma, sarcomatoid carcinoma, inflammatory myofibroblastic tumor, and various sarcomas.^[7]^ Imaging examinations, including US, CT, and magnetic resonance (MR) are used for assessing mediastinal SFTs. It is not possible to determine whether a mass is benign or malignant by imaging examination. However, The CT findings can reveal the extent of tumor, regional invasion, and vascular encasement calcification. To our best knowledge, Positron emission tomography (PET)-CT was a new tool for assessing the SFTs. Suehisa et al^[8]^ reported a case of mediastinal SFT that assessed with PET-CT, it showed weak uptake by the tumor, with a maximum standardized uptake value (max SUV) of 1.85. Definite diagnosis is base on immunohistochemistry. CD34, which is a hematopoietic progenitor antigen, plays a substantial role in supporting the diagnosis of SFTs. Recently, some studies have shown positivity for Bcl-2, CD99, and vimentin are important markers for the diagnosis of an SFT, but it not considered specific.^[9]^

The most successful management of mediastinal SFTs is by surgical resection, which can be either radical or staged.^[5]^ In the past, the majority of mediastinal tumor excisions have been approached via standard median sternotomy. With the improvement of thoracoscopic surgical skills, surgery is increasing being performed by thoracoscope. The present patient was not considered to be a suitable candidate for video-assisted thoracic surgery (VATS) because the tumor was malignant and huge.

Mediastinal malignant SFTs can recur after complete resection. With the local recurrence, second surgery is remains considered the best method.^[7]^ The prognosis for patients with mediastinal malignant is unknown. Xue et al^[7]^ reported a rare case of mediastinal SFT invading the lung and diaphragm. The patient remains alive with no sign of recurrence more than 18 months after her operation.^[7]^ Zhang et al^[3]^ reported 13 cases of mediastinal SFT that managed by surgery. The patients had no recurrence or metastasis, except one died of cerebral hemorrhage 2 months after the surgery. But in our present study, there was metastasis in the chest wall and right middle lung after 2 months follow-up postoperation, and patient was dead after 4 months.

In conclusion, we described a rare case of 42-year-old male who presented with a mediastinal malignant SFT invading the lung and pericardium, and received tumor complete excision. The patient recovered uneventful and discharged on day 13. After 2 months follow-up postoperation, there was metastasis in the chest wall and right middle lung. The patient refused any treatment and was dead after 4 months. Due to the high mortality, new strategy is needed to monitor the rapid metastasis of this type of tumor.

## Acknowledgment

We would like to acknowledge the patient for allowing this case to be published.
